# Alvascience: A New Software Suite for the QSAR Workflow Applied to the Blood–Brain Barrier Permeability

**DOI:** 10.3390/ijms232112882

**Published:** 2022-10-25

**Authors:** Andrea Mauri, Matteo Bertola

**Affiliations:** Alvascience Srl, Via Giuseppe Parini, 35, 23900 Lecco, Italy

**Keywords:** quantitative structure–activity relationship, machine learning, data curation, molecular descriptors, model validation, model deployment, blood–brain barrier permeability

## Abstract

Quantitative structure–activity relationship (QSAR) and quantitative structure–property relationship (QSPR) are established techniques to relate endpoints to molecular features. We present the Alvascience software suite that takes care of the whole QSAR/QSPR workflow necessary to use models to predict endpoints for untested molecules. The first step, data curation, is covered by alvaMolecule. Features such as molecular descriptors and fingerprints are generated by using alvaDesc. Models are built and validated with alvaModel. The models can then be deployed and used on new molecules by using alvaRunner. We use these software tools on a real case scenario to predict the blood–brain barrier (BBB) permeability. The resulting predictive models have accuracy equal or greater than 0.8. The models are bundled in an alvaRunner project available on the Alvascience website.

## 1. Introduction

**Figure 1:**
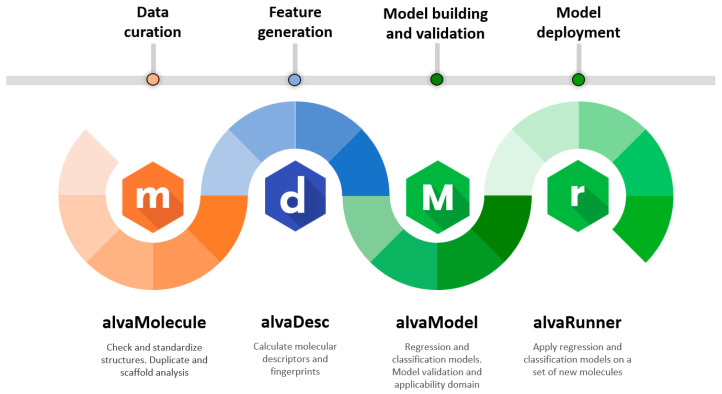
QSAR workflow using Alvascience suite.

Assessing the effects of a large number of chemical substances is becoming increasingly necessary in modern society. Along with in vivo and in vitro approaches, in silico is considered as a solution to help while dealing with a huge number of novel molecules [1]. In silico methods are a set of strategies that allow the use of computers to study the properties and behavior of chemical compounds. These methods include quantitative structure–activity relationship (QSAR) and quantitative structure–property relationship (QSPR), which can relate a certain endpoint such as pharmacological activity, biological toxicity, physicochemical property, and environmental variable with features of chemical compounds such as molecular descriptors and fingerprints. Therefore, QSAR/QSPR models help in the prediction of even untested chemicals endpoints, and they do that by starting from the molecular structure information alone. The increasing interest in these techniques, over the past decades, is shown both by the increase in scientific publications [2,3,4] and by the use of QSAR/QSPR in legislation and regulatory practices. Key examples of the latter are the OECD principles for the validation of (Q)SAR models [5] proposed in 2004 by the Organization for Economic Co-operation and Development (OECD) and the Registration, Evaluation, Authorisation and Restriction of Chemicals (REACH) regulation by the European Union. These regulations also show that particular attention is given to a clear process to develop QSAR/QSPR models starting from the definition of the endpoint, the use of known algorithms, and the ability to measure the goodness-of-fit, robustness and predictivity of the models. In order to fulfill the need to develop QSAR/QSPR models in a logical manner, also compliant to the above-mentioned regulations, this paper presents a process in the form of a workflow (Figure 1). The main steps of the workflow such as data curation, feature generation, model building and validation and model deployment are covered by the software tools comprised in the Alvascience’s software suite. In this paper, we introduce the Alvascience software suite by applying its tools to a real case study. The case identified is related to the blood–brain barrier (BBB), which regulates the entrance of substances to the central nervous system (CNS) [6]. As a result, we present a set of models to predict the BBB permeability, which was built and validated with Alvascience tools.

### 1.1. Overview of the Alvascience Software Suite

The Alvascience suite is comprised of four software programs. The interaction of these software programs is described in the workflow in Figure 1. Each step of the workflow relates to a specific topic and the software program used to deal with it. Although these programs have been designed to work according to the described workflow, they can also be used independently from each other. In fact, each of them is a standalone software provided with a graphical user interface (GUI) and available for Windows, Linux and macOS. To facilitate the integration with existing systems, some software programs are also equipped with a command line interface (CLI) and interfaces for Python and KNIME [7].

The QSAR process starts from a molecular dataset. The curation of such dataset is a recommended step, and every aspect of it can be taken care of by using alvaMolecule. With alvaMolecule, the user can perform all expected activities such as aromatization, standardization, scaffold and duplicate analysis, and checking for anomalies. The input and output of alvaMolecule is a simple molecular file written in the most common molecular file formats.

Feature generation is the step where descriptors and fingerprints are calculated for each molecule of the dataset. This step is taken care by alvaDesc [8]. With almost 6000 descriptors, alvaDesc can characterize the molecules with a set of informative features. Some analytic tools (such as PCA and t-SNE [9,10]) are present to perform preliminary valuation on the generated descriptors. The molecules and their features can be saved in an alvaDesc project. Such a project will become the input of the next step tool, alvaModel. However, the features can also be exported in common formats to be used with third-party programs.

Building and validating models is the core of QSAR. Using alvaModel, the user can generate regression and classification models. The models can be built either by manually selecting the features or by using Genetic Algorithms [11] to search for the best features automatically. Each model can be validated by using standard regression or classification scores (e.g., R^2^ or Accuracy). The validation can be either internal (i.e., using cross-validation with scores such as the Q^2^) or external (i.e., using the training and test set) [12]. The validation phase allows choosing the best models which can then be exported into an alvaRunner project.

The deployment of models is a step of the QSAR workflow which is often overlooked. Making the models available and usable by colleagues and other researchers is important [13]. To tackle this issue, Alvascience developed alvaRunner, which can apply models on a new molecular dataset. The user of alvaRunner does not have to deal with the feature generation or dependencies of the models, since alvaRunner takes care of everything and displays the models’ results. The applicability domain, if present, is shown next to each model result to help to attest if the prediction is reliable.

### 1.2. Data Curation

Data curation is the active management of data from its collection to a careful consideration of its format and content. In particular, chemical data curation entails taking care of the molecular structures and information, such as endpoints, associated with each molecule. It is a key element of the QSAR workflow, and it should be the first step, since without a mindful data curation, descriptor generation and model building can be negatively influenced [14]. The curation of molecular data can be one of the most time-consuming phases of the model building process; it often requires human expertise to check molecular structures, even manually, to identify potential problems. To ease this difficult task, Alvascience developed alvaMolecule, which is a desktop software program that performs all the actions needed to curate a molecular dataset. Its graphical user interface also allows the visualization and analysis of the molecules contained in the imported files. Different molecular file formats are supported such as SMILES, MDL/SDF, and MOL2.

**Figure 2:**
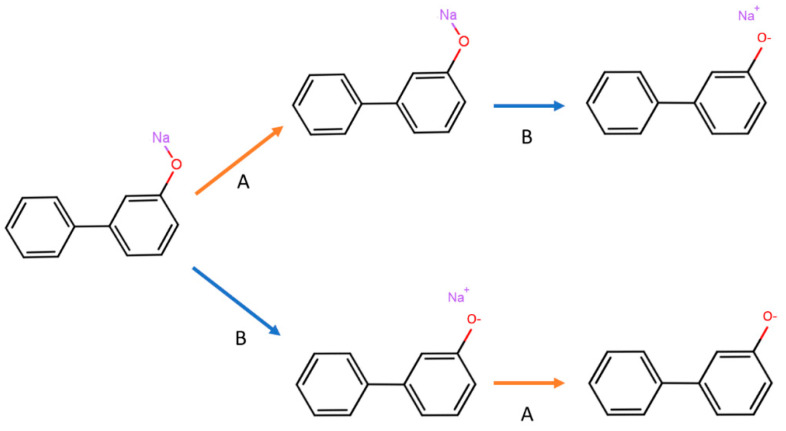
Same standardizers applied to the same molecule (Sodium 1,1’-biphenyl-3-olate) in a different order yield different results. “Remove monoatomic fragments” is represented with arrow A (orange) and “Convert unusual covalent bonds to ionic bonds” is represented with arrow B (blue).

Chemical data curation is often presented as a strict sequence of tasks to perform [15]. This approach can be helpful to clarify and organize operations to be completed to ensure that a molecular dataset is ready to be used. However, it also has some drawbacks, since a rigid ordered step-by-step procedure is not always the approach that yields the desired result (Figure 2). Therefore, it is advisable to check each task of the data curation manually. The researcher, using alvaMolecule, can move freely from the different phases of data curation and even repeat the same task in different moments if needed. A common example is with the Check structures feature, which allows finding anomalies in the molecular structures. Usually, this is the first task to undertake when working with a new dataset because it gives an idea of which type of problems must be addressed. It can also be useful to check the structures again at the end of the data curation to make sure that all issues have been resolved. Among the many controls that can be performed (Table 1), there is the possibility of flagging molecules containing multiple structures, unusual valence, charged atoms, unusual aromaticity representation, and other peculiar characteristics. Particular attention must be paid to the aromatization of molecules, since it is common to find the same molecule represented with different or even incorrect aromatic rings. Additionally, various cheminformatics tools can handle the aromaticity differently. Therefore, using alvaMolecule is recommended to make the representation of aromatic rings uniform (i.e., Kekulé or aromatic form) before starting with the checks.

**Figure 3:**
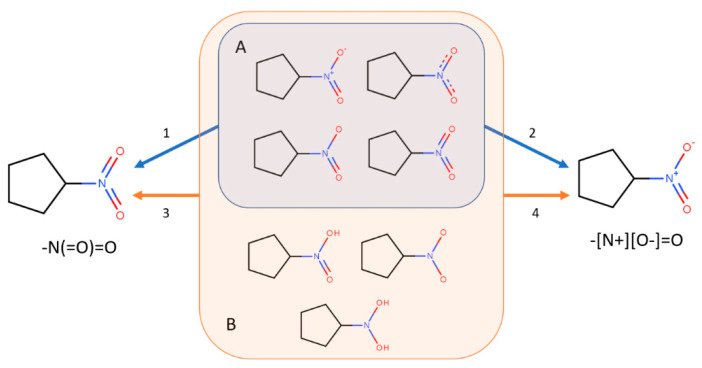
Standardization of the nitro group for nitrocyclopentane-like molecules. Group A (blue) shows molecules that can be standardized using the predefined standardizers (blue arrows). Arrow 1 shows the effect of the “Standardize nitro group (-N(=O)=O)” standardizer. Arrow 2 shows the effect of “Standardize nitro group (-[N+][O-]=O)”. Group B (orange) includes the molecules standardized using a custom standardizer (orange arrows). Arrow 3 shows the effect of the custom standardizer defined as “[N;D3:1](~[O;D1:2])(~[O;D1:3])>>[*+0;H0:1](=[*+0;H0:2])=[*+0;H0:3]”. Arrow 4 shows the effect of the custom standardizer defined as “[N;D3:1](~[O;D1:2])(~[O;D1:3])>>[*+1;H0:1](=[*+0;H0:2])-[*−1;H0:3]”.

A common request in chemical data curation is removing molecular structures having undesired characteristics such as mixture, salts, organometallic and inorganic compounds [15]. This can become a mandatory task when working with software tools that are not able to calculate molecular descriptors for such types of chemical structures. Even if this is not the case for alvaDesc, which can handle organometallic compounds and has different techniques to deal with disconnected structures, alvaMolecule can be used to remove molecules having these undesired characteristics. Checking and manually removing molecules might not always be enough. Therefore, the molecular standardizers provided by alvaMolecule can be used to fix erroneous molecular representation, add or remove specific features or standardize specific structural features (Table 1). For example, using the nitro group standardizer, one can convert the nitro group from the original representation to a nitrogen atom connected to two oxygen atoms by two double bonds independently (Figure 3).

Identifying duplicated molecules can be crucial, since it is also a well-documented issue [16,17] of many publicly available datasets. Knowing if two molecules are the same molecule is not necessarily a straightforward problem. In fact, it can depend on their representation and on the characteristics used to compared them. By using alvaMolecule, it is possible to control which parameters to keep under consideration while performing the duplicate identification. Parameters such as the stereochemistry can affect this process, yielding different results (Table 2).

Each molecule can be characterized by a set of properties either already included in the molecular dataset or calculated by alvaMolecule. The former ones are additional fields read by alvaMolecule directly from the molecule files and organized in the molecular worksheet. The latter ones are a minimal subset of the descriptors calculated by alvaDesc, and they include some basic physicochochemical properties and drug-like indexes. They can be used to perform preliminary analysis such as showing the distribution of a given property in the dataset. Such analysis is part of manual inspection of the molecules that is recommended for chemical data curation. The researcher can sort and filter the molecules by their properties using the alvaMolecule worksheet, which also allows the removal of molecules and editing their imported properties. A set of charts is provided to help visualize and select the molecules with a certain property range. Furthermore, each molecule, or one having a similar structure, can be searched for in public databases such as PubChem [18] and Google Patents/Scholar. Finding a molecule in public databases can also be useful for retrieving information related to the compound (e.g., IUPAC name).

### 1.3. Feature Generation

Molecular fingerprints and descriptors are used to describe molecules in numerical terms [19,20,21]. Their calculation involves mathematical and algorithmic manipulation of the molecule that can be performed using specific software tools, such as alvaDesc. Using alvaDesc version 2, the user can calculate several types of fingerprints and almost 6000 descriptors. It calculates MACCS 166 fingerprint [22] and Extended Connectivity Fingerprints (ECFP) [21] which can be tuned with a set of parameters (e.g., maximum fragment size). The descriptors are grouped in different blocks so that the user can also choose to calculate a subset of them (Table 3). A common property used to characterize descriptors is dimensionality [23,24]. Each descriptor can be said to have one of the following dimensions: 0D, 1D, 2D or 3D. The zero-dimensional descriptors are the ones calculated without considering the connections between the atoms. The one-dimensional descriptors consider only a part of the entire molecule topology. The two-dimensional descriptors use the whole molecule graph. The three-dimensional descriptors are calculated using the 3D coordinates of the molecule. Special attention must be used when dealing with 3D descriptors, as the same molecule can have many possible 3D conformations. Therefore, 3D descriptors can be heavily influenced by the 3D conformer used to obtain the molecule coordinates.

Preliminary analysis of the molecular datasets and the calculated descriptors can be conducted using alvaDesc functionalities. Different plots can be used to graphically represent the data. In addition, a more global idea of the data can be formed by using Principal Component Analysis (PCA) and the t-SNE. User-friendly graphical interfaces help the user to navigate through the different options. The analysis can also involve external variables such as molecular endpoints or other descriptors. These external variables can be imported using a text-based file (e.g., CSV file). Instead, the calculated fingerprints and descriptors can be exported in tabbed text-file so that they can be used by other tools that are not part of the Alvascience suite. During this phase, the number of saved descriptors can be reduced by undergoing a variable reduction. The variable reduction analyzes the data based on the option selected by the user (e.g., a standard deviation below a certain value) and removes all the descriptors that do not respect the specified requirements. Even though exporting the descriptors is possible, saving an alvaDesc project is recommended to preserve all the molecular and calculated data. The alvaDesc project achieves two goals: it allows the re-opening of the data for future analysis and it can be used for model building in the following tool of the Alvascience suite workflow, alvaModel.

The graphical user interface of alvaDesc is what most of users need, but in some cases, it may be necessary to integrate alvaDesc in existing workflow. For this purpose, alvaDesc is equipped with a CLI that can be invoked from scripts or other software technologies such as KNIME and Python. A KNIME node was specifically designed to simplify the integration with alvaDesc. In addition, Alvascience developed a Python module, called alvaDescCLIWrapper, to allow developers to take advantage of alvaDesc calculations through a simple programming interface.

### 1.4. Model Building and Validation

The step of model building and validation is performed in the Alvascience workflow by using alvaModel. With this tool, a researcher can perform all the necessary actions to create, select and validate models for the given data in accordance with the OECD principles [5]. These principles were defined as guidelines to facilitate the consideration of a QSAR model for regulatory purposes. The OECD principles are:

1. A defined endpoint;
2. An unambiguous algorithm;
3. A defined domain of applicability;
4. Appropriate measures of goodness-of-fit, robustness and predictivity;
5. A mechanistic interpretation, if possible.

The starting point of alvaModel is an alvaDesc project. Such a project can be imported and transformed into an alvaModel project which will be the container of all the generated models. Three elements are required to generate models in alvaModel: the molecules, the molecule features (e.g., the descriptors) and at least one endpoint. The latter, also known as the target variable, must be defined before building the model in accordance with the first OECD principle. The molecules and their features are always present in the original project, but the target variable can be missing. In this case, the target variable must be imported using the import external variables feature from a text file (e.g., a CSV file). One of the first steps, before proceeding with the model building, is to split the dataset into training and test sets. Splitting the dataset allows for an external validation on the test set. The splitting can be performed using a specifically designed interface that allows the user to split randomly, following a rule (e.g., venetian blinds) or using the value of some other variable.

An important distinction between problems that can be tackled by machine learning models is regression and classification. Regression problems are about predicting a quantity, while classification problems deal with the prediction of a discrete or categorical class. Both types of problems can be dealt with by alvaModel. In fact, alvaModel calculates several regression (e.g., linear regression (OLS) and Partial Least Squares (PLS)) and classification (e.g., Linear and Quadratic Discriminant Analysis (LDA/QDA) and K-Nearest Neighbors (KNN)) models. All the available models, in accordance with the second OECD principle, are based on well-known techniques and algorithms. It is also possible to predict an endpoint by combining the predictions of two or more models by building a consensus model. The consensus model uses a function; for example, in case of regression, it takes the average of the selected models to output the final prediction [25,26]. 

The models can be built in alvaModel either by starting the manual or automatic mode. The manual mode allows the user to manually select the features to be used in the model. This mode does not involve a variable selection, since the user decides each of the model descriptors. It is particularly useful when a known model must be reproduced. In contrast, the idea behind the automatic mode is that given the large number of descriptors that alvaDesc calculates, it can be challenging to find a good subset of features to train your model with. The automatic mode, also called automatic model generation, uses Genetic Algorithms to perform a series of feature selections searching for the best combination of features among the entire set of features [11]. The Genetic Algorithms take inspiration from Darwinian theory assuming that only the best fitted members of a population survive, and new members can appear by mutating and recombining their genes. The population is composed by models, and their fitness is measured using a score. Both the manual and automatic mode are managed by a step-by-step user interface (i.e., a wizard) which guides the user through all the possible choices. One of the steps of the wizard allows performing a variable reduction in the selected descriptors. This is usually completed to reduce the sheer number of descriptors eliminating the ones that are either constant, quasi-constant, or too similar to each other [27]. In addition, alvaModel allows defining the policy to handle missing values by either deciding to remove molecules or features containing a missing value.

**Figure 4:**
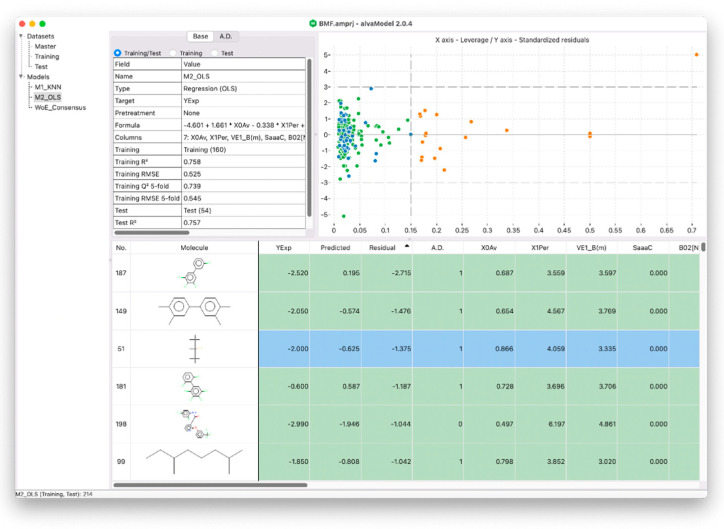
The graphical user interface of alvaModel showing a regression model with its parameters, metrics (scores), training and test molecules and their predicted and input values represented both with a grid and with a Williams plot.

The third OECD principle states the importance of calculating an applicability domain which represents the theoretical region of the chemical space where a model can generate reliable predictions [28,29,30]. This can be completed in alvaModel, for example, by calculating distance-based applicability domains which measure the distance between a sample molecule and the model training set and determines if the sample is inside the applicability domain based on a threshold. Another technique, known as leverage applicability domain, estimates the distance from the model’s experimental space using the leverage matrix, which is also used in the Williams plot (Figure 4). In fact, the Williams plot can be useful for graphically detecting outliers that are outside the leverage applicability domain [12].

In accordance with the fourth OECD principle, alvaModel provides a set of tools and scores to attest the goodness-of-fit, robustness and predictivity of models. The scores are numeric metrics that can be used to measure the quality of both regression (e.g., R^2^) [31] and classification (e.g., Accuracy) models. Their use is part of the practice to determine the ability of the model to represent a behavior or a real phenomenon called model validation. A class of specific scores is the one based on the cross-validation (e.g., Q^2^ for regression models), which is a technique to test the model’s ability to predict new data that was not used in the training phase. Another useful tool for model validation is the Y-randomization [32].

**Figure 5:**
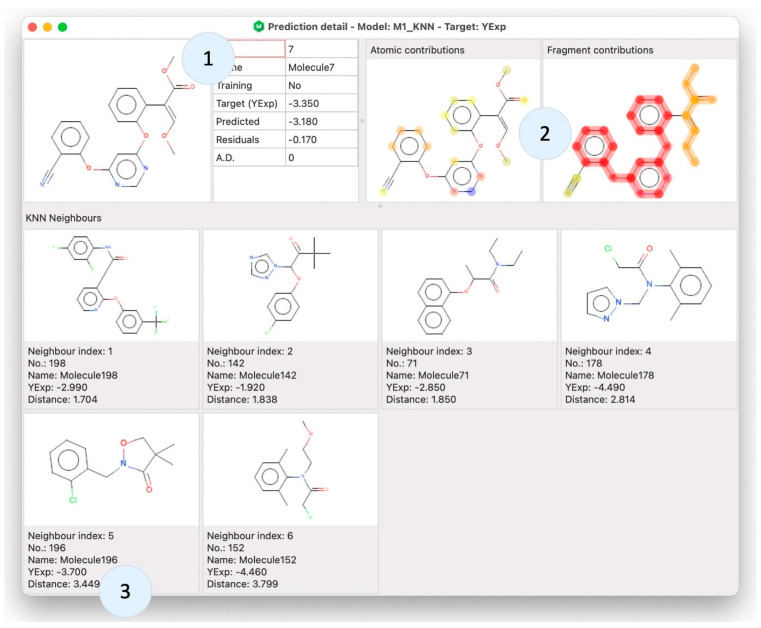
Prediction detail window comprises three sections. The first one (1) shows the target information, the second one (2) includes the atomic and fragment contributions and the third one (3) includes the KNN neighbors (for KNN models).

Once the model is created, gaining knowledge about the prediction of a specific sample molecule is often required. This can lead, in accordance with the fifth OECD principle, to an interpretation of the model behavior. By using alvaModel Prediction detail, it is possible to show information about a single molecule in connection with a model (Figure 5). For example, it is possible to check the neighbors of a molecule in a KNN model and the atomic [33] and fragment contributions [34]. These two are visual representations of the contribution in the model prediction of the atoms, frameworks and side chains of the selected sample molecule.

**Figure 6:**
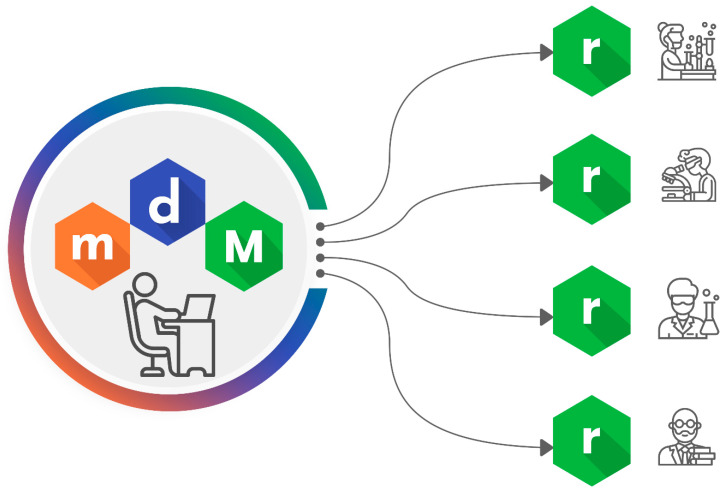
Once generated, models can be deployed as alvaRunner projects. Colleagues and other researchers can make predictions on their molecules using only alvaRunner.

Once the models are built and validated, they can be packaged in an alvaRunner project. This project can be opened using alvaRunner, which is the last step in the Alvascience suite workflow (Figure 6). 

### 1.5. Model Deployment

The researcher’s job often stops at the previous step where the model is built and validated. In fact, passing a model to other researchers, colleagues or making it available online can be a difficult task. This is so because, for example, making the model available may not be enough for a user to apply the model to a new set of molecules. The model may require dependencies or the exact version of a set of tools that may not be available for all users. The absence of these prerequisites may invalidate the possibility of reproducing the researcher’s work. To address this need, Alvascience developed alvaRunner. Without any prior knowledge or the need for extra tools, a researcher can use alvaRunner to predict the endpoint defined in an alvaRunner project for a given molecular dataset (Figure 6). The internal engine of alvaRunner makes all the necessary calculations for the predicting process. The user only needs to open two files: the alvaRunner project and the file containing the molecules. With these two files, alvaRunner interprets the molecules, calculates the necessary descriptors and fingerprints, applies the expected pretreatments and finally applies the models to predict the target values. An alvaRunner project can contain many models on a single endpoint. Each model can be associated with an applicability domain so that the alvaRunner user can determine if the prediction of a molecule can be considered reliable or not. 

The results are shown in a handy grid that allows to sort and filter the data. The results can also be exported to a tabbed text file or popular molecular formats such as SMILES and MDL to be used elsewhere. Similarly to alvaModel, alvaRunner has a dedicated user interface that shows information about a single molecule prediction. This can be helpful to gain some insights into the behavior of the model for the selected molecule.

In addition to a graphical user interface, alvaRunner has a CLI which can be used directly from a shell or integrated in a user workflow. Such CLI can also be invoked by KNIME using the node specifically developed by Alvascience.

## 2. Results and Discussion

We experimented with different model types, parameters and features for BBB prediction. We selected three classification models (M1, M2, M3) based on KNN and one consensus model (MC). In this section, we present the proposed models describing the features used and their performances (Table 4). 

### 2.1. Model M1

M1 is a KNN model using the MACCS 166 fingerprints as features. The KNN is a non-parametric method often used for classification. In the training phase, the features and target values of the training dataset are stored inside the model. The prediction is performed by considering the classes of the k closest molecules (i.e., neighbors) of the training dataset. The MACCS 166 fingerprint is a fixed size structural key comprising a dictionary of 166 well-defined molecular features [22]; every bit of the MACCS 166 fingerprint indicates the presence or absence of a specific molecular feature.

### 2.2. Model M2

M2 is a KNN model using the Extended Connectivity Fingerprints (ECFP) as features. The ECFP [21], also known as a circular fingerprint, is a hashed fingerprint obtained by systematically enumerating all circular fragments grown radially from each non-hydrogen atom of the molecule up to the set radius (also known as maximum length). The maximum length chosen for M2 was 3, which corresponds to an ECFP6, since this fingerprint is often described with the diameter of the circular fragment. The ECFP can also be characterized by its size, which for M2 was chosen to be 2048. 

### 2.3. Model M3

M3 is a KNN model using nine descriptors as features. The features were standardized to ensure that different scales did not affect the Euclidean distance measurement. This model was found by using the Automatic model generation of alvaModel where the maximum number of descriptors was set to 10. This number was chosen to simplify the interpretability of the resulting model. The more features there are (other authors use more than 100 descriptors [35,36]), the more difficult it is to interpret the model [12].

The molecular descriptors included in the model provide different kinds of information useful to discriminate between BBB+ and BBB- molecules. A brief description of the selected descriptors is provided:

1. Mp is the mean atomic polarizability (scaled on Carbon atom); this molecular descriptor is a 0-dimensional descriptor and belongs to the constitutional indices block. Constitutional descriptors are the simplest molecular descriptors, since they only provide information on molecule composition and not on how the atoms are connected. Mp is calculated as the average value of the atomic polarizability, and it is calculated considering also the hydrogen atoms included in the molecule. Since atomic polarizabilities for hydrogen, fluorine, oxygen and nitrogen atoms are lower than for chlorine, sulfur, bromine, phosphorus and iodine, Mp increases for molecules having a lower rate of saturated bonds (i.e., when the number of hydrogen atoms decreases) and when the percentage of atoms belonging to the second group increases. This descriptor is not influenced by the size of the molecule but only by its atomic composition. Specifically, it can be able to discriminate between molecules according to their atomic polarizabilities.
2. nN is another zero-dimensional descriptor belonging to the constitutional indices block and corresponds to the number of nitrogen atoms included in the molecule. The training set includes 2997 over a total of 3525 molecules having at least one nitrogen atom. The number of nitrogen atoms included in the training set ranges from 0 to 20.
3. MPC07 is the molecular path count of order 7 and belongs to the walk and path counts logical block. A path is a walk without any repeated vertex, the path count of order 7 is the total number of paths of length 7 in the molecular graph. Since molecular path counts may be very large for molecules with a large number of atoms, alvaDesc calculates this descriptor as the logarithm of the molecular path count. Molecular path counts provide information both on the size and complexity of the molecules [37,38].
4. NssssN+ is a one-dimensional descriptor belonging to the atom-type E-state indices [39,40] and represents the number of quaternary ammonium cations included in the molecule [41]. The training set includes 61 molecules having at least one quaternary ammonium cation.
5. SHED_DL is a SHannon Entropy Descriptor considering donor (D) and lipophilic (L) atoms. SHED descriptors are derived from the distributions of potential pharmacophore points (PPP) in the molecule [42,43]. SHED descriptors are calculated as the Shannon entropy [44] and can be used to quantify the variability in a feature–pair distribution [45]. SHED_DL will assume values equal to 1 for molecules where all donor–lipophilic atom pairs are at the same topological distance and values near to 20 for molecules where donor–lipophilic atom pairs topological distances are uniformly distributed.
6. SHED_AN is another SHannon Entropy Descriptor but considering acceptor (A) and negative (N) atoms. Analogously to SHED_DL, this descriptor provides information related to the distribution of acceptor and negative atoms in the molecule. Molecules having all the acceptor–negative atom pairs at the same topological distance will have value equal to 1, while for molecules where acceptor and negative atoms are at different topological distances, SHED_AN will tend to 20.
7. F09[C-C] is a frequency atom pair descriptor which counts all the atom pairs of carbon atoms at a topological distance equal to 9. F09[C-C] values, as well as MPC07 values, grow with the molecule size and complexity but, unlike MPC07 values, only consider the carbon atoms.
8. TPSA(Tot) is the topological polar surface area of a molecule [46] and is defined as the sum of the surfaces of all polar atoms. Polar surface area has been used in medicinal chemistry for the optimization of a drug’s capability to permeate cells [47,48], and it is considered an important descriptor to evaluate the blood–brain barrier penetration [35,49].
9. MLOGP2 is the square value of MLOGP, where MLOGP is the Moriguchi octanol-water partition coefficient model [50,51]. MLOGP2 is a frequently used descriptor in QSAR model for the prediction of blood–brain barrier permeability [35].

### 2.4. Model MC

MC is a consensus model based on M1, M2 and M3 (also referred to as contained models). It predicts the target variable by first using the contained models and then assigning the class based on the majority of results [25,26]. The idea of the consensus model is based on the concept that the combination of more models can improve the overall reliability of the prediction. Consensus models have been used in many QSAR studies [52,53,54,55,56,57,58]. 

The applicability domain (AD) of the consensus model was automatically calculated, by alvaModel, as the conjunction of the contained models’ active ADs. In this way, a molecule is considered within the consensus model’s AD only if it is within the ADs of all the contained models. More than 95% of the training set molecules are within the AD of MC, showing a certain homogeneity of the molecules. The AD can still be helpful for future use of the model. In fact, by using alvaRunner, it is possible to verify the AD values of each of the models for new molecules that need to be predicted. Predictions that fall outside the AD are less likely to be reliable.

## 3. Materials and Methods

A recent study [59] about the blood–brain barrier (BBB) was selected to apply the predictive capabilities of the Alvascience software suite. The BBB is the term used to describe the ability of the central nervous system to block access to structures that circulate in the bloodstream. The BBB permeability is a relevant endpoint for drug discovery when designing compounds to target or avoid the CNS [60]. Many models for BBB permeation prediction have been developed over the years [35,36,61] also because computational methods are considered an interesting alternative to the experimental determination, which is difficult and expensive [62]. The selected study, to the best of our knowledge, presented the biggest dataset related to BBB containing 7807 molecules gathered from 50 published resources. All the molecules contain a label accounting for their permeability of the BBB (BBB+ or BBB-). Such label was determined, when not present in the original sources, by using a logBB threshold of −1. The next sections describe how data were curated and which features (descriptors and fingerprints) were generated. Finally, it is described how binary classification models to predict BBB permeability were built.

### 3.1. Data Curation—alvaMolecule

The original dataset comprises 7807 molecules; 7 of these were eliminated due to issues with their PubChem CID. The remaining 7800 molecules were kekulized using alvaMolecule to avoid issues related to different aromaticity representations, since they were taken from different sources and therefore possibly treated with different cheminformatics tools. Using Check structures functionality, a few molecules with potential issues were identified and removed. Molecules with multiple structures can be handled by alvaDesc, but they can reduce the number of descriptors that can be calculated; therefore, 10 molecules with disconnected structures were removed. Atoms having unusual valence must be checked carefully; using alvaMolecule, 6 molecules containing such atoms were removed (Table 5). Finally, 4 molecules containing only one atom were eliminated (e.g., Kr). 

Next, an analysis of the duplicated structures was performed by ignoring the stereochemistry because we decided to only use descriptors that are not affected by the molecular stereochemistry. The duplicate analysis yielded 1486 groups of duplicated molecules for a total of 5252 duplicated molecules. Despite some studies [63,64] suggesting that BBB permeability is influenced by the stereochemistry, only 130 groups contained molecules having the same structure, except for stereochemistry, but different BBB values. The molecules of such groups were removed from the dataset. For the groups of molecules having duplicated structure and the same BBB value, only one molecule per group was retained. 

The final dataset comprises 3884 molecules having a number of atoms ranging from 2 to 276. In addition, the scaffold analysis [65], performed with alvaMolecule, showed the presence of 1936 different scaffolds and 223 molecules with no rings. That shows a certain variability in the dataset. The most common scaffolds are benzene and cyclopentaphenanthrene-like (Table 6).

### 3.2. Feature Generation—alvaDesc

The dataset is composed of molecules represented using the SMILES format [66] which does not carry any 3D information. Therefore, the 3D descriptors were not calculated. A total of 4179 descriptors were calculated using alvaDesc. In addition, the Extended Connectivity Fingerprints (ECFP) [21] and MACCS 166 fingerprint [22] were determined. 

### 3.3. Model Building—alvaModel

By using alvaModel, the dataset was split into training and test including in the former the molecules having a value in the PubChem CID column (3525) and in the latter the remaining molecules (359). Both training and test sets are unbalanced in favor of the BBB+ class (Table 7). The binary classification models were built, and their quality was attested by using known metrics such as Sensitivity, Specificity and Accuracy also by cross-validation.

## 4. Conclusions

In this work, we presented a software suite capable of handling the needs of a QSAR workflow. We demonstrated the ability of each software tool by applying them to a binary classification QSAR problem according to the OECD principles. We started from the data curation with alvaMolecule. The feature generation was taken care of by calculating fingerprints and descriptors with alvaDesc. Then, we developed four models to predict the ability of molecules to pass the blood–brain barrier by using alvaModel. The models have been validated with well-known metrics, cross-validation and on an external test set. Finally, we bundled these models into a project which can be opened in alvaRunner to allow the prediction of the BBB permeability on new molecules without any extra software tool. The prediction is also equipped, for each of the models, with an applicability domain. The alvaRunner project is available at https://www.alvascience.com/model-blood-brain-barrier-bbb-permeability/ (accessed on 16 October 2022).

## Figures and Tables

**Table 1:** List of checkers and standardizers available in alvaMolecule.

Check Structures
Multiple structures
Unusual valence
Covalent/Ionic bond
Total charge
Isotope
Charged atom
None carbon atoms
Non-standard atom set (H,B,C,N,O,P,S,F,Cl,Br,I)
Aromaticity
Radical atom
**Standardizers**
Convert unusual covalent bonds to ionic bonds
Add charge to quaternary nitrogen
Remove exceeding hydrogens
Add missing hydrogens
Remove monoatomic fragments
Retain biggest fragment
Standardize nitro group (-N(=O)=O)
Standardize nitro group (-[N+][O-]=O)
Standardize azide group
Standardize diazo group
Clear isotopes
Clear chirality
Clear bond direction
Remove radicals
Remove hydrogens
Neutralize atoms
Neutralize molecule
Custom standardizer

**Table 2:** Examples of how the duplicate analysis can be influenced by the chosen parameters. The duplicate analysis can be influenced by the chosen parameters. For example, molecules with PubChem CIDs 5726 and 6,327,751 are considered equal (and therefore duplicates) if the formal charge and the number of hydrogens are ignored. Instead, molecules with PubChem CID 676,643 and 24,066 are considered duplicates if the stereochemistry is ignored.

Mol	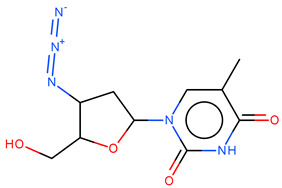	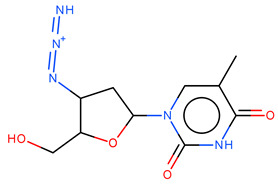
CID	5726	6,327,751
SMILES	O=C(O)c1cc(N=Nc2ccc(S(=O)(=O)Nc3ccccn3)cc2)ccc1O	O=C(O)c1cc(N=Nc2ccc(S(=O)(=O)Nc3ccccn3)cc2)ccc1O
Mol	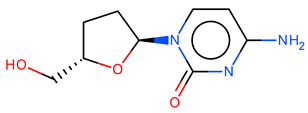	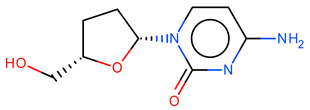
CID	676,643	24,066
SMILES	Nc1ccn([C@@H]2CC[C@@H](CO)O2)c(=O)n1	Nc1ccn([C@H]2CC[C@@H](CO)O2)c(=O)n1

**Table 3:** List of alvaDesc descriptors blocks.

Block Name	Descriptors	Block Name	Descriptors
Constitutional indices	50	WHIM descriptors	114
Ring descriptors	35	GETAWAY descriptors	273
Topological indices	79	Randić molecular profiles	41
Walk and path counts	46	Functional group counts	154
Connectivity indices	37	Atom-centered fragments	115
Information indices	51	Atom-type E-state indices	346
2D matrix-based descriptors	608	Pharmacophore descriptors	165
2D autocorrelations	213	2D Atom Pairs	1596
Burden eigenvalues	96	3D Atom Pairs	36
P_VSA-like descriptors	69	Charge descriptors	15
ETA indices	40	Molecular properties	27
Edge adjacency indices	324	Drug-like indices	30
Geometrical descriptors	38	CATS 3D descriptors	300
3D matrix-based descriptors	132	WHALES descriptors	33
3D autocorrelations	80	MDE descriptors	19
RDF descriptors	210	Chirality descriptors	70
3D-MoRSE descriptors	224		

**Table 4:** Performance of the models. The distances J.T and E. stand for Jaccard Tanimoto and Euclidean, respectively. The cross-validation (CV) is 5-fold.

			Training Set				CV	Test Set		
Model	k	Distance	Sensitivity	Specificity	Accuracy	Balanced Accuracy	Accuracy	Sensitivity	Specificity	Accuracy
M1	5	J.T.	0.879	0.662	0.801	0.771	0.798	0.755	0.910	0.822
M2	5	J.T.	0.902	0.619	0.800	0.761	0.796	0.775	0.890	0.825
M3	5	E.	0.898	0.637	0.804	0.767	0.799	0.775	0.871	0.816
MC	-	-	0.917	0.645	0.819	0.781	0.814	0.760	0.916	0.827

**Table 5:** Example of a molecule identified using the ‘Unusual valence’ checker. The molecule from the original dataset has a carbon atom with a missing hydrogen. Its name and PubChem CID correspond to two different molecules.

Source	Original Dataset	PubChem	PubChem
Molecule	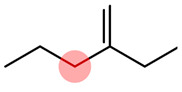	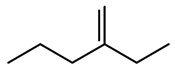	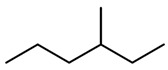
Name	3-methylhexane	2-Ethyl-1-pentene	3-Methylhexane
CID	520,668	520,668	11,507
SMILES	C=C([CH]CC)CC	CCCC(=C)CC	CCCC(C)CC
Chemical formula	C7H13	C7H14	C7H16

**Table 6:** Examples of scaffolds identified in the dataset. The most frequent scaffold is the benzene, being the scaffold of 236 molecules; additionally, 5 cyclopentaphenanthrene scaffolds are shown with the number of molecules containing them.

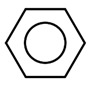	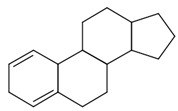	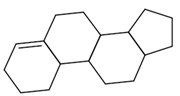
Molecules: 236	Molecules: 92	Molecules: 37
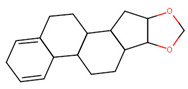	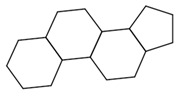	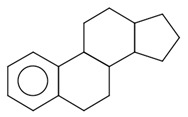
Molecules: 17	Molecules: 11	Molecules: 7

**Table 7:** Number of molecules in the training and test sets also grouped by class.

Dataset	Total	BBB+	BBB−
Training	3525	2257	1268
Test	359	204	155

## Data Availability

The alvaRunner project containing the BBB permeability models is available at https://www.alvascience.com/model-blood-brain-barrier-bbb-permeability/ (accessed on 16 October 2022). The curated dataset is available in the Supplementary Materials. The software tools presented in the paper were developed by Alvascience (https://www.alvascience.com (accessed on 16 October 2022)): alvaMolecule (v2.0.0, https://www.alvascience.com/alvamolecule/ (accessed on 16 October 2022)), alvaDesc (v2.0.12, https://www.alvascience.com/alvadesc/ (accessed on 16 October 2022)), alvaModel (v2.0.4, https://www.alvascience.com/alvamodel/ (accessed on 16 October 2022)), andalvaRunner (v2.0.4, https://www.alvascience.com/alvarunner/ (accessed on 16 October 2022)).

## Supplementary Materials

The following supporting information can be downloaded at: https://www.mdpi.com/article/10.3390/ijms232112882/s1.

## References

[1] Benfenati E., Gini G., Hoffmann S., Luttik R. (2010). Comparing in Vivo, in Vitro and in Silico Methods and Integrated Strategies for Chemical Assessment: Problems and Prospects. ATLA Altern. Lab. Anim..

[2] Willett P. (2020). The Literature of Chemoinformatics: 1978–2018. Int. J. Mol. Sci..

[3] Yousefinejad S., Hemmateenejad B. (2015). Chemometrics Tools in QSAR/QSPR Studies: A Historical Perspective. Chemom. Intell. Lab. Syst..

[4] Li L., Hu J., Ho Y.-S. (2014). Global Performance and Trend of QSAR/QSPR Research: A Bibliometric Analysis. Mol. Inform..

[5] European Commission Environment Directorate General (2014). Guidance Document on the Validation of (Quantitative) Structure-Activity Relationship [(Q)SAR] Models.

[6] Daneman R., Prat A. (2015). The Blood–Brain Barrier. Cold Spring Harb. Perspect. Biol..

[7] Berthold M.R., Cebron N., Dill F., Gabriel T.R., Kötter T., Meinl T., Ohl P., Sieb C., Thiel K., Wiswedel B., Preisach C., Burkhardt H., Schmidt-Thieme L., Decker R. (2008). KNIME: The Konstanz Information Miner. Data Analysis, Machine Learning and Applications.

[8] Mauri A., Roy K. (2020). AlvaDesc: A Tool to Calculate and Analyze Molecular Descriptors and Fingerprints. Ecotoxicological QSARs.

[9] Hinton G., Roweis S. (2003). Stochastic Neighbor Embedding. Advances in Neural Information Processing Systems 15 (NIPS 2002).

[10] Van der Maaten L., Hinton G. (2008). Visualizing Data Using T-SNE. J. Mach. Learn. Res..

[11] Leardi R. (2007). Genetic Algorithms in Chemistry. J. Chromatogr. A.

[12] Gramatica P., Reisfeld B., Mayeno A.N. (2013). On the Development and Validation of QSAR Models. Alternatives to Laboratory Animals: ATLA.

[13] Nantasenamat C., Roy K. (2020). Best Practices for Constructing Reproducible QSAR Models. Ecotoxicological QSARs.

[14] Alves V.M., Auerbach S.S., Kleinstreuer N., Rooney J.P., Muratov E.N., Rusyn I., Tropsha A., Schmitt C. (2021). Curated Data In—Trustworthy In Silico Models Out: The Impact of Data Quality on the Reliability of Artificial Intelligence Models as Alternatives to Animal Testing. Altern. Lab. Anim..

[15] Fourches D., Muratov E., Tropsha A. (2010). Trust, But Verify: On the Importance of Chemical Structure Curation in Cheminformatics and QSAR Modeling Research. J. Chem. Inf. Model..

[16] Hähnke V.D., Kim S., Bolton E.E. (2018). PubChem Chemical Structure Standardization. J. Cheminform..

[17] Monge A., Arrault A., Marot C., Morin-Allory L. (2006). Managing, Profiling and Analyzing a Library of 2.6 Million Compounds Gathered from 32 Chemical Providers. Mol. Divers..

[18] Kim S., Chen J., Cheng T., Gindulyte A., He J., He S., Li Q., Shoemaker B.A., Thiessen P.A., Yu B. (2021). PubChem in 2021: New Data Content and Improved Web Interfaces. Nucleic Acids Res..

[19] Todeschini R., Consonni V. (2009). Molecular Descriptors for Chemoinformatics.

[20] Mauri A., Consonni V., Todeschini R. (2017). Molecular Descriptors. Handbook of Computational Chemistry.

[21] Rogers D., Hahn M. (2010). Extended-Connectivity Fingerprints. J. Chem. Inf. Model..

[22] Durant J.L., Leland B.A., Henry D.R., Nourse J.G. (2002). Reoptimization of MDL Keys for Use in Drug Discovery. J. Chem. Inf. Comput. Sci..

[23] Grisoni F., Ballabio D., Todeschini R., Consonni V. (2018). Molecular Descriptors for Structure-Activity Applications: A Hands-On-Approach. Computational Toxicology.

[24] Todeschini R., Consonni V. (2000). Handbook of Molecular Descriptors.

[25] Baurin N., Mozziconacci J.C., Arnoult E., Chavatte P., Marot C., Morin-Allory L. (2004). 2D QSAR Consensus Prediction for High-Throughput Virtual Screening. An Application to COX-2 Inhibition Modeling and Screening of the NCI Database. J. Chem. Inf. Comput. Sci..

[26] Valsecchi C., Grisoni F., Consonni V., Ballabio D. (2020). Consensus versus Individual QSARs in Classification: Comparison on a Large-Scale Case Study. J. Chem. Inf. Model..

[27] Ballabio D., Consonni V., Mauri A., Claeys-Bruno M., Sergent M., Todeschini R. (2014). A Novel Variable Reduction Method Adapted from Space-Filling Designs. Chemom. Intell. Lab. Syst..

[28] Netzeva T.I., Worth A.P., Aldenberg T., Benigni R., Cronin M.T.D., Gramatica P., Jaworska J.S., Kahn S., Klopman G., Marchant C.A. (2005). Current Status of Methods for Defining the Applicability Domain of (Quantitative) Structure-Activity Relationships. Altern. Lab. Anim..

[29] Sahigara F., Mansouri K., Ballabio D., Mauri A., Consonni V., Todeschini R. (2012). Comparison of Different Approaches to Define the Applicability Domain of QSAR Models. Molecules.

[30] Gadaleta D., Mangiatordi G.F., Catto M., Carotti A., Nicolotti O. (2016). Applicability Domain for QSAR Models. Int. J. Quant. Struct. Relatsh..

[31] Alexander D.L.J., Tropsha A., Winkler D.A. (2015). Beware of R 2: Simple, Unambiguous Assessment of the Prediction Accuracy of QSAR and QSPR Models. J. Chem. Inf. Model..

[32] Tropsha A., Gramatica P., Gombar V.K. (2003). The Importance of Being Earnest: Validation Is the Absolute Essential for Successful Application and Interpretation of QSPR Models. QSAR Comb. Sci..

[33] Riniker S., Landrum G.A. (2013). Similarity Maps—A Visualization Strategy for Molecular Fingerprints and Machine-Learning Methods. J. Cheminform..

[34] Polishchuk P.G., Kuz’min V.E., Artemenko A.G., Muratov E.N. (2013). Universal Approach for Structural Interpretation of QSAR/QSPR Models. Mol. Inform..

[35] Zhang L., Zhu H., Oprea T.I., Golbraikh A., Tropsha A. (2008). QSAR Modeling of the Blood-Brain Barrier Permeability for Diverse Organic Compounds. Pharm. Res..

[36] Shaker B., Yu M.S., Song J.S., Ahn S., Ryu J.Y., Oh K.S., Na D. (2021). LightBBB: Computational Prediction Model of Blood-Brain-Barrier Penetration Based on LightGBM. Bioinformatics.

[37] Randić M., Brissey G.M., Spencer R.B., Wilkins C.L. (1979). Search for All Self-Avoiding Paths for Molecular Graphs. Comput. Chem..

[38] Randić M. (1979). Characterization of Atoms, Molecules, and Classes of Molecules Based on Paths Enumeration. MATCH Commun. Math. Comput. Chem..

[39] Hall L.H., Mohney B., Kier L.B. (1991). The Electrotopological State: An Atom Index for QSAR. Quant. Struct. Relatsh..

[40] Hall L.H., Kier L.B. (1995). Electrotopological State Indices for Atom Types: A Novel Combination of Electronic, Topological, and Valence State Information. J. Chem. Inf. Comput. Sci..

[41] Zheng G., Zhang Z., Lockman P.R., Geldenhuys W.J., Allen D.D., Dwoskin L.P., Crooks P.A. (2010). Bis-Azaaromatic Quaternary Ammonium Salts as Ligands for the Blood–Brain Barrier Choline Transporter. Bioorg. Med. Chem. Lett..

[42] Schneider G., Neidhart W., Giller T., Schmid G. (1999). “Scaffold-Hopping” by Topological Pharmacophore Search: A Contribution to Virtual Screening. Angew. Chem. Int. Ed..

[43] Renner S., Fechner U., Schneider G., Langer T., Hoffmann R.D. (2006). Alignment-Free Pharmacophore Patterns—A Correlation-Vector Approach. Pharmacophores and Pharmacophore Searches.

[44] Shannon C.E., Weaver W. (1949). The Mathematical Theory of Communication.

[45] Gregori-Puigjané E., Mestres J. (2006). SHED: Shannon Entropy Descriptors from Topological Feature Distributions. J. Chem. Inf. Model..

[46] Ertl P., Rohde B., Selzer P. (2000). Fast Calculation of Molecular Polar Surface Area as a Sum of Fragment-Based Contributions and Its Application to the Prediction of Drug Transport Properties. J. Med. Chem..

[47] Pajouhesh H., Lenz G.R. (2005). Medicinal Chemical Properties of Successful Central Nervous System Drugs. NeuroRx.

[48] Hitchcock S.A., Pennington L.D. (2006). Structure-Brain Exposure Relationships. J. Med. Chem..

[49] Gupta M., Lee H.J., Barden C.J., Weaver D.F. (2019). The Blood-Brain Barrier (BBB) Score. J. Med. Chem..

[50] Moriguchi I., Hirono S., Liu Q., Nakagome I., Matsushita Y. (1992). Simple Method of Calculating Octanol/Water Partition Coefficient. Chem. Pharm. Bull..

[51] Moriguchi I., Hirono S., Nakagome I., Hirano H. (1994). Comparison of Reliability of Log P Values for Drugs Calculated by Several Methods. Chem. Pharm. Bull..

[52] Ballabio D., Biganzoli F., Todeschini R., Consonni V. (2016). Qualitative Consensus of QSAR Ready Biodegradability Predictions. Toxicol. Environ. Chem..

[53] Asturiol D., Casati S., Worth A. (2016). Consensus of Classification Trees for Skin Sensitisation Hazard Prediction. Toxicol. Vitr..

[54] Abdelaziz A., Spahn-Langguth H., Schramm K.-W., Tetko I.V. (2016). Consensus Modeling for HTS Assays Using In Silico Descriptors Calculates the Best Balanced Accuracy in Tox21 Challenge. Front. Environ. Sci..

[55] Falcón-Cano G., Molina C., Cabrera-Pérez M.A. (2020). ADME Prediction with KNIME: In Silico Aqueous Solubility Models Based on Supervised Recursive Machine Learning Approaches. ADMET DMPK.

[56] Mamada H., Nomura Y., Uesawa Y. (2021). Prediction Model of Clearance by a Novel Quantitative Structure–Activity Relationship Approach, Combination DeepSnap-Deep Learning and Conventional Machine Learning. ACS Omega.

[57] Grisoni F., Consonni V., Vighi M. (2018). Acceptable-by-Design QSARs to Predict the Dietary Biomagnification of Organic Chemicals in Fish. Integr. Environ. Assess. Manag..

[58] Cassotti M., Consonni V., Mauri A., Ballabio D. (2014). Validation and Extension of a Similarity-Based Approach for Prediction of Acute Aquatic Toxicity towards Daphnia Magna. SAR QSAR Environ. Res..

[59] Meng F., Xi Y., Huang J., Ayers P.W. (2021). A Curated Diverse Molecular Database of Blood-Brain Barrier Permeability with Chemical Descriptors. Sci. Data.

[60] Abbott N.J. (2004). Prediction of Blood–Brain Barrier Permeation in Drug Discovery from in Vivo, in Vitro and in Silico Models. Drug Discov. Today Technol..

[61] Kumar R., Sharma A., Alexiou A., Bilgrami A.L., Kamal M.A., Ashraf G.M. (2022). DeePred-BBB: A Blood Brain Barrier Permeability Prediction Model With Improved Accuracy. Front. Neurosci..

[62] Toropov A.A., Toropova A.P., Beeg M., Gobbi M., Salmona M. (2017). QSAR Model for Blood-Brain Barrier Permeation. J. Pharmacol. Toxicol. Methods.

[63] Chang K.L., Pee H.N., Yang S., Ho P.C. (2015). Influence of Drug Transporters and Stereoselectivity on the Brain Penetration of Pioglitazone as a Potential Medicine against Alzheimer’s Disease. Sci. Rep..

[64] Fong C.W. (2015). Permeability of the Blood–Brain Barrier: Molecular Mechanism of Transport of Drugs and Physiologically Important Compounds. J. Membr. Biol..

[65] Bemis G.W., Murcko M.A. (1996). The Properties of Known Drugs. 1. Molecular Frameworks. J. Med. Chem..

[66] Weininger D. (1988). SMILES, a Chemical Language and Information System. 1. Introduction to Methodology and Encoding Rules. J. Chem. Inf. Model..

